# Veritas® bovine pericardium for immediate breast reconstruction: a xenograft alternative to acellular dermal matrix products

**DOI:** 10.1007/s00238-012-0736-9

**Published:** 2012-06-17

**Authors:** Mehrdad Mark Mofid, Michael S. Meininger, Martin S. Lacey

**Affiliations:** 1 Division of Plastic Surgery, University of California San Diego, 4150 Regents Park Row Suite #300, La Jolla, CA 92037 USA; 2 Division of Plastic Surgery, Wayne State University, 1080 Kirts Blvd Suite #700, Troy, MI 48084 USA; 3 Department of Plastic and Hand Surgery, University of Minnesota, Minneapolis, 640 Jackson Street, Mail Stop 11503B, St. Paul, MN 55101 USA

**Keywords:** Veritas, Bovine pericardium, Acellular dermal matrix, Breast reconstruction, Tissue, Expander, Breast complications

## Abstract

**Background:**

The technical advantages in utilizing human acellular dermal matrix (ADM) products as pectoral extenders in immediate breast reconstruction with tissue expanders or implants are well documented in the medical literature. In this study, the authors examine a commonly used biologic xenograft product that has not yet been described in the medical literature for use in immediate breast reconstruction to determine whether a lower overall complication rate is identified compared to published data on ADM products.

**Methods:**

A retrospective multicenter medical record review of data on 54 subjects in 93 tissue expander/implant-based, consecutive, immediate breast reconstructions from three surgeons at different institutions was performed in which Veritas® bovine pericardium was used as the biologic graft material for the pectoral extender.

**Results:**

Over a 24-month period with an average of 11-month follow-up, complication rates using Veritas® in breast reconstruction for seroma formation (7.5 %), marginal skin flap necrosis (5.4 %) infection (6.5 %), and capsular contracture (0 %) were found to compare equally or favorably with statistically significant lower overall complications relative to one comparison study and lower rates of marginal skin flap necrosis relative to two comparison studies based upon previously published data from multisurgeon studies using ADM products.

**Conclusions:**

Overall complications were found to be lower with Veritas® than ADM products in comparable multisurgeon studies, though this was found to be statistically significant in only one comparison study.

Level of Evidence: Level II, theraputic study.

## Introduction

There has been nearly a decade of experience with biologic graft material to assist with implant-based, post-mastectomy breast reconstruction and revisionary breast surgery [1]. The reported benefits of utilizing grafts as pectoral extenders include the ability to add greater tissue expander volume at the time of surgery and decreased postsurgical tissue expansion, facilitated direct to implant reconstruction, improved inframammary fold definition and decreased rates of capsular contracture [2–6]. To date, the vast majority of published reports have chronicled the experience with allograft and xenograft acellular dermal matrix (ADM) products for breast reconstruction and for revisionary breast surgery. Abundant outcomes data comparing complication rates utilizing ADM products and conventional submuscular placement of tissue expanders is available [7–12].

Despite the reported technical benefits of using ADM products for breast reconstruction, several large multisurgeon studies comparing conventional tissue expander reconstruction with those involving ADM grafts have documented increased rates of seroma formation, mastectomy skin flap necrosis, infection, and overall complications [8–10]. In an effort to maintain the technical benefits of breast reconstruction using biologic grafts as pectoral extenders, a commonly used, but not yet studied, material for use in breast reconstruction, Veritas® bovine pericardium (Synovis Surgical Innovations, St. Paul, MN, USA), a non-ADM xenograft, was evaluated in tissue expander and implant-based breast reconstruction. This study was performed to determine whether noted advantages using ADM products, such as ease of use, ease of setting the inframammary fold, and low rates of capsular contracture could be achieved with lower rates of seroma formation, infection, marginal skin flap necrosis, and overall complications relative to ADM products. An extensive history of use with noncross-linked bovine pericardium for abdominal wall, urogynecologic, neurosurgical, and cardiothoracic reconstruction has been well established, suggesting that it may be an ideal biocompatible material for reconstructive and revisionary breast surgery [13–16].

## Materials and methods

A multicenter, retrospective medical record review was performed for consecutive Veritas® patients who underwent mastectomy with immediate breast reconstruction between 2009 and 2010 at three different medical institutions by three different surgeons (MMM, MSM, and MSL). The study method and design were reviewed and approved by the Western Institutional Review board and Health Partners Institutional Review board. A total of 54 subjects yielding data on 93 consecutive tissue expander/implant-based immediate breast reconstructions were included over the 24-month period. Patients meeting the inclusion criteria were over the age of 18 and underwent mastectomy with immediate breast reconstruction using Veritas® collagen matrix. Patients who underwent delayed breast reconstruction, reconstruction with a biologic graft other than Veritas®, or patients with concurrent autologous flap reconstruction were excluded from the study. Data on age, body mass index, use of prophylactic antibiotics, mastectomy weight, duration of surgical drainage, cancer stage, chemotherapy and radiation history, presence of comorbidities, including diabetes, hypertension, cardiac disease, and smoking history were tabulated.

The duration of postsurgical expansion prior to implant exchange and all complications including seroma formation, hematoma, marginal skin flap necrosis, infection, capsular contracture, and need for tissue expander or implant removal resulting in failure of the reconstructive effort were analyzed. Seromas were subdivided into minor seromas requiring only office-based drainage and major seromas requiring operative drain placement. Infections were also subdivided into major infections requiring inpatient hospitalization and/or implant removal and minor infections requiring outpatient oral antibiotic treatment. Bilateral mastectomies were recorded as two separate cases.

**Fig. 1:**
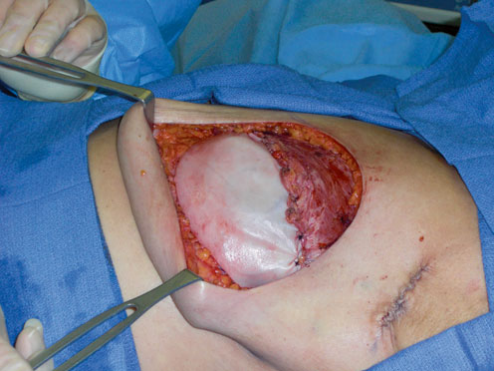
Intraoperative placement of a 6- × 18-cm sheet of Veritas® secured to the caudal edge of the pectoralis major muscle, inframammary fold, and serratus anterior muscle over a tissue expander with 2–0 polydioxanone sutures.

Prophylactic intravenous antibiotic use and intraoperative antibiotic irrigation with bacitracin solution for tissue expanders and implants was recorded for all patients. The surgical technique utilized entailed submuscular dissection of the pectoralis major muscle, suturing of the Veritas® bovine pericardium to the inframammary fold, caudal edge of the pectoralis muscle, and the serratus anterior muscle laterally with 2–0 polydiaxone suture, thus enveloping the tissue expander/implant with Veritas® for inferior pole coverage. Tissue expanders were filled intraoperatively to allow for minimal skin tension on mastectomy skin flaps (Fig. 1). One or two drains above the pectoralis major muscle and exiting the axilla were used on all patients.

For statistical analysis, a logistic regression was used to perform univariate analyses on each patient demographic and comorbidity to determine if a relationship exists with the occurrence of any study complication. Any potential risk factor that rendered a *p* < 0.10 was retained for a subsequent multivariate analysis. Patient demographics, comorbidities, and all complication rates were compared with previously published data of multisurgeon clinical studies using ADM products and control studies with no biologic and total submuscular tissue expander coverage.

## Results

Over a 24-month period, a total of 54 patients underwent 93 immediate breast reconstructive procedures with tissue expanders or implants. Three plastic surgeons performed reconstructive procedures at three different medical institutions utilizing a standardized technique for breast reconstruction with Veritas® bovine pericardium as a pectoral extender. There were no significant differences in patient characteristics between surgeons, including age, comorbidities, smoking status, radiation and chemotherapy history, cancer stage, body mass index, or mastectomy specimen weight. The mean period of follow-up was 11 months. Pre- or postoperative radiation therapy was recorded in 16.7 % of cases. Chemotherapy was administered postoperatively in 27.8 % of subjects and preoperatively in 3.7 % of subjects. In addition, 25.9 % of subjects were former smokers, and 3.7 % were present smokers. Postsurgical drains were placed for an average of 10.1 days. In patients receiving tissue expanders, the average duration to implant exchange was 20.6 weeks. Eighteen patients (33.3 %) underwent single-stage reconstruction with silicone implants in one or both breasts, 34 patients (63 %) of patients underwent two-stage reconstruction with a tissue expander and Veritas® at the first stage and a silicone implant at the second stage, and 2 patients (3.7 %) underwent a single-stage reconstruction in one breast and a two-stage reconstruction in the other breast. This represented a total of 36 single stage reconstructions (38.7 %) and 57 two-stage reconstructions (61.3 %) (Table 1).

**Table 1:** Patient demographics

Patient characteristics	Veritas (*n* = 54 subjects, 93 breasts)
Subjects per surgeon (%)	
Site 1	15 (27.8 %)
Site 2	30 (55.6 %)
Site 3	9 (16.7 %)
Mean age, years ± SD	50.5 ± 10.1
Mean BMI ± SD	24.6 ± 4.7
Mean mastectomy specimen weight in grams ± SD	452.5 ± 290.7
Diabetes (%)	2 (3.4 %)
Hypertension (%)	12 (22.2 %)
Carotid artery disease (%)	1 (1.9 %)
History of smoking (%)	
Current smoker	2 (3.7 %)
Former smoker	14 (25.9 %)
Non smoker	38 (70.4 %)
Chemotherapy (%)	17 (31.5 %)
Radiation therapy (%)	9 (16.7 %)
Subjects with single-stage reconstruction (%)	18 (33.3 %)
Subjects with two-stage reconstruction (%)	34 (63.0 %)
Subjects with single-/two-stage reconstruction (%)	2 (3.7 %)
Breasts with single-stage reconstruction (%)	36 (38.7 %)
Breasts with two-stage reconstruction (%)	57 (61.3 %)
Mean duration of drain placement, days ± SD	10.1 ± 6.0

**Fig. 2:**
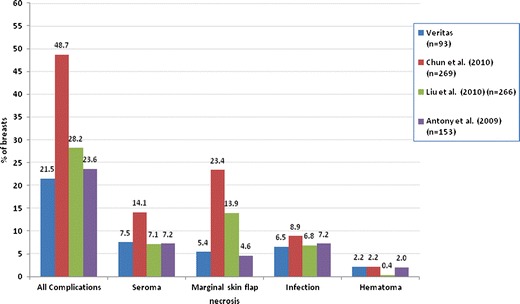
Bar graph of complication rates with Veritas® in immediate breast reconstruction as compared to AlloDerm® from previously published data in multisurgeon studies [8–10]

The seroma rate with Veritas® was 7.5 % with a 1.1 % major seroma rate and a 6.4 % minor seroma rate. The cumulative infection rate was 6.5 % with a 2.1 % major infection rate and 4.3 % minor infection rate. Only two cases of culture-specific microorganisms were identified in the study, with one patient found to have *Pseudomonas aeruginosa* and another patient a staphylococcal organism. Both of these patients required implant removal. Marginal skin flap necrosis was noted in 5.4 % of patients. There were no reported cases of capsular contracture in the study over the 11-month follow-up period. The presence of all complications was 21.5 % (Table 2, Fig. 2). At least one complication was noted in 17 breasts (18.3 %). There were no significant differences among complication rates or demographics in the study between tissue expander and single-stage implant reconstructions or in complication rates between surgeons in the study.

**Table 2:** Comparison of complication rates with Veritas® in immediate breast reconstruction with AlloDerm® (previously published data) in multi-surgeon studies

Complication	Veritas® (*n* = 93)	Chun et al. [8] AlloDerm (*n* = 269)	Lui et al. [10] AlloDerm (*n* = 266)	Antony et al. [9] AlloDerm (*n* = 153)
		*p* value	*p* value	*p* value
All complications by breast (%)	20 (21.5 %)	131 (48.7 %)	75 (28.2 %)	36 (23.6 %)
		*p* < 0.0001	*p* = 0.222	*p* = 0.756
≥1 complication by breast (%)	17 (18.3 %)	–	–	–
Seroma (%)	7 (7.5 %)	38 (14.1 %)	19 (7.1 %)	11 (7.2 %)
		*p* = 0.104	*p* = 1.000	*p* = 1.000
Major	1 (1.1 %)	–	–	–
Minor	6 (6.4 %)	–	–	–
Marginal skin flap necrosis (%)	5 (5.4 %)	63 (23.4 %)	37 (13.9 %)	7 (4.6 %)
		*p* < 0.0001	*p* = 0.026	*p* = 0.769
Infection (%)	6 (6.5 %)	24 (8.9 %)	18 (6.8 %)	11 (7.2 %) (includes cellulitis)
		*p* = 0.521	*p* = 1.000	*p* = 1.000
Major	2 (2.1 %)	22 (8.2 %)	13 (4.9 %)	–
		P = 0.052	P = 0.371	
Minor	4 (4.3 %)	2 (0.7 %)	5 (1.9 %)	–
		*p* = 0.040	*p* = 0.245	
Hematoma (%)	2 (2.2 %)	6 (2.2 %)	1 (0.4 %)	3 (2.0 %)
		1.000	0.166	1.000
Capsular contracture	0 (0 %)	–	–	–

## Discussion

Many plastic surgeons have adopted the use of acellular dermal matrix products in reconstructive and revisionary breast surgery due to the technical advantages in setting the inframammary fold, increased intraoperative fill volumes of tissue expanders, the shorter duration of the expansion process, facility in performing single-stage direct to implant reconstructions, and the increasing body of evidence showing lower documented capsular contracture rates [17]. The enthusiasm for the use of ADMs has been tempered by the higher reported rates of seroma formation, peri-prosthetic infection, and mastectomy skin flap necrosis [8–10].

The ideal graft material for use in tissue expander/implant breast reconstruction incorporates all of the advantages that have been heretofore noted with ADM products but minimizes complication rates and has a favorable relative cost of product [18, 19]. In this study, the authors have determined that relative to other multisurgeon published studies on ADM products in breast reconstruction, Veritas® bovine pericardium exhibits a lower overall complication rate of 21.5 %, though this was found to be statistically significant only in comparison to one other study by Chun et al. (48.7 %, *p* < 0.0001) [8]. No statistical significance of overall complication rates was found relative to two other multisurgeon studies by Liu et al. and Antony et al. [9, 10] Lower rates of infection and seroma were not found to be statistically significant. A lower rate of marginal skin flap necrosis (5.4 %) was found to be statistically significant relative to Chun et al. [8] (23.4 %, *p* < 0.0001) and Liu et al. [10] (13.9 %, *p* = 0.026). Veritas® was found to have a zero reported rate of capsular contracture over the follow up period in the study.

Given the vast majority of seromas, infections and mastectomy skin flap necrosis are identified within the first several months after the performance of immediate breast reconstruction after mastectomy, the relatively short follow-up period of 11 months is believed to adequately represent a true picture of the overall complication rate relative to ADM products. With respect to capsular contracture, though it represents a progressive phenomenon, Prantl et al. found that 58 % of all contractures occurred within the first 11 months after implantation, 17 % within 3 years and 25 % after 5 years [20]. It is possible that the clinical findings associating low capsular contracture rates with ADM products, which are thought to be related to the decrease in inflammatory mediators, myofibroblasts, fibroblast cellularity, and foreign body giant cell reaction, may also be applicable to noncross-linked bovine pericardium.

Other innate properties of noncross-linked bovine pericardium may also explain the favorable complication rate noted in this study relative to one other multisurgeon, multicenter clinical study. As a thinner product [21], Veritas® has been found, in histological and biomechanical abdominal wall studies, to have superior capacity for host revascularization relative to ADM products. In an incisional hernia study by Deeken et al. in the pig model, at 1 year, no differences in strength or stiffness of the repair site were noted between AlloDerm® and Veritas®. At 1, 6, and 12 months, however, Veritas® scored significantly higher than AlloDerm® in histological analyses of remodeling with greater host cellular infiltration, fewer inflammatory cells, higher levels of extracellular matrix deposition, greater neovascularization, and markedly less fibrous encapsulation [22]. It is also possible that the lower rates of marginal skin flap necrosis noted by the authors of this study may be related to a less zealous approach to intraoperative expansion of the tissue expanders relative to surgeons in other studies.

From a technical standpoint, the lower elastin content of bovine pericardium (2.98 %) relative to ADM products (5–7 %) [21] may be an advantage in maximizing the soft tissue coverage afforded by the pectoralis major muscle and in preventing the window shading of the caudal muscle edge during the expansion process. Products with higher elastin content such as AlloDerm® may be more prone to stretch, exhibit less stiffness, and experience greater deformation in response to force displacement [13].

As the cost of biologic graft materials is becoming increasingly important, given the economic constraints of the provision of reconstructive breast procedures and health care in general, the cost per square centimeter of Veritas® bovine pericardium compares favorably to AlloDerm®. A 6- × 18-cm sheet of Veritas® has a list price of $3,024 or $28.00/cm^2^. A 6- × 16-cm “thick” AlloDerm® sheet has a list price of $3,562 or $37.10 cm^2^ [23] This represents a 25 % reduction in price relative to a similar sized sheet of AlloDerm®.

Despite these advantages identified with Veritas® bovine pericardium for immediate breast reconstruction, properties inherent to the thin nature of the material make it less attractive for use than ADM products for the specific purpose of camouflaging the rippling and wrinkling noted in secondary breast reconstruction or in aesthetic revisionary breast surgery. The increased thickness afforded with thick or ultrathick ADM products provide a higher degree of soft tissue augmentation over palpable or visible implants, especially in the upper pole [24, 25]. The greatest degree of soft tissue interposition in implant-based breast reconstruction is afforded by the submuscular placement of expanders and implants in the upper pole, though, at times, additional augmentation of the soft tissue envelope is possible with autologous fat grafting or adjunctive placement of biologic graft material in secondary procedures.

## Conclusion

The authors present the first multicenter, multisurgeon, retrospective study of Veritas® bovine pericardium for use in immediate reconstruction as a biologic graft pectoral extender after mastectomy using tissue expanders or implants. Overall complications were found to be lower with Veritas® than ADM products in comparable multisurgeon studies though this was found to be statistically significant in only one comparison study. A zero rate of capsular contracture over 11 months following implantation is noted. A lower overall cost of product is noted relative to AlloDerm®. Additional studies are needed to confirm these findings and to determine whether the extracellular mechanisms responsible for seroma formation, infection, skin flap necrosis, and capsular contracture are in fact relatively modulated in response to the materials tested.
